# Risk factors and electrocardiogram characteristics for mortality in critical inpatients with COVID‐19

**DOI:** 10.1002/clc.23492

**Published:** 2020-10-22

**Authors:** Lingzhi Li, Shudi Zhang, Bing He, Xiaobei Chen, Shihong Wang, Qingyan Zhao

**Affiliations:** ^1^ Department of Cardiology Renmin Hospital of Wuhan University Wuhan China; ^2^ Department of Pediatrics Renmin Hospital of Wuhan University Wuhan China; ^3^ Department of Neurosurgery Renmin Hospital of Wuhan University Wuhan China; ^4^ Department of Infectious Diseases Renmin Hospital of Wuhan University Wuhan China; ^5^ Cardiovascular Research Institute Wuhan University Wuhan China; ^6^ Hubei Key Laboratory of Cardiology Wuhan University Wuhan China

**Keywords:** arrhythmia, COVID‐19, critical type, mortality, risk factors

## Abstract

**Background:**

The novel severe acute respiratory syndrome coronavirus 2 (SARS‐CoV‐2) has spread worldwide.

**Hypothesis:**

The possible risk factors that lead to death in critical inpatients with coronavirus disease 2019 (COVID‐19) are not yet fully understood.

**Methods:**

In this single‐center, retrospective study, we enrolled 113 critical patients with COVID‐19 from Renmin Hospital of Wuhan University between February 1, 2020 and March 15, 2020. Patients who survived or died were compared.

**Results:**

A total of 113 critical patients with COVID‐19 were recruited; 50 (44.3%) died, and 63 (55.7%) recovered. The proportion of patients with ventricular arrhythmia was higher in the death group than in the recovery group (*P* = .021) and was higher among patients with myocardial damage than patients without myocardial damage (*P* = .013). Multivariate analysis confirmed independent predictors of mortality from COVID‐19: age > 70 years (HR 1.84, 95% CI 1.03‐3.28), initial neutrophil count over 6.5 × 10^9^/L (HR 3.43, 95% CI 1.84‐6.40), C‐reactive protein greater than 100 mg/L (HR 1.93, 95% CI 1.04‐3.59), and lactate dehydrogenase over 300 U/L (HR 2.90, 95% CI 1.26‐6.67). Immunoglobulin treatment (HR 0.39, 95% CI 0.21‐0.73) can reduce the risk of death. Sinus tachycardia (HR 2.94, 95% CI 1.16‐7.46) and ventricular arrhythmia (HR 2.79, 95% CI 1.11‐7.04) were independent ECG risk factors for mortality from COVID‐19.

**Conclusions:**

Old age (>70 years), neutrophilia, C‐reactive protein greater than 100 mg/L and lactate dehydrogenase over 300 U/L are high‐risk factors for mortality in critical patients with COVID‐19. Sinus tachycardia and ventricular arrhythmia are independent ECG risk factors for mortality from COVID‐19.

**Abbreviations**
ARDS: acute respiratory distress syndrome
BNP: B‐type natriuretic peptide
COPD: chronic obstructive pulmonary disease
COVID‐19: coronavirus disease 2019
ECG: electrocardiogram
IMV: invasive mechanical ventilation
MOF: multiple organ failure
NIV: noninvasive ventilation
NP: nucleocapsid protein
ORF: gene and open reading frame
SARS‐CoV‐2: novel severe acute respiratory syndrome coronavirus 2

## 1 INTRODUCTION

At present, there are outbreaks of the novel severe acute respiratory syndrome coronavirus 2 (SARS‐CoV‐2), and this virus has spread throughout the world. Common clinical manifestations in patients include fever, nonproductive cough, dyspnea, myalgia, fatigue, normal or decreased leukocyte counts, and radiographic evidence of pneumonia.^1^ Although most patients are thought to have a favorable prognosis, older patients, and those with chronic underlying conditions may have worse outcomes. Patients with severe illness may develop dyspnea and hypoxemia within 1 week after the onset of the disease, which may quickly progress to acute respiratory distress syndrome (ARDS) or multiple organ failure (MOF).^2^ A previous study reported that the mortality was 62% among critically ill patients with coronavirus disease 2019 (COVID‐19) in Wuhan.^3^ However, possible risk factors leading to poor clinical outcomes have not been well delineated.

Fever, hypoxemia, and myocardial injury caused by COVID‐19 can easily cause arrhythmia.^4^, ^5^ In a cohort of patients with COVID‐19, Wang observed that arrhythmias were present in 16.7% of patients.^6^ However, due to the lack of strict epidemiological investigations, the occurrence, and outcomes of arrhythmia in patients with COVID‐19 are still unclear. In the present study, we aimed to investigate risk factors and electrocardiogram (ECG) characteristics for mortality in critical inpatients with COVID‐19.

## 2 METHODS

### 2.1 Patient selection

This single‐center, retrospective, observational study was performed at Renmin Hospital of Wuhan University (Wuhan, China). A total of 113 critical inpatients with COVID‐19 from February 1, 2020 to March 15, 2020 were enrolled. All patients were confirmed to have COVID‐19 by performing RT‐PCR on samples from the respiratory tract. The diagnosis of COVID‐19 was based on the WHO interim guidelines.^7^ All patients met the clinical criteria for critical‐type COVID‐19. Critical‐type COVID‐19 was defined based on the New Coronavirus Pneumonia Prevention and Control Program in China (sixth edition).^8^ Patients who met one of the following criteria were considered to have critical‐type COVID‐19: respiratory failure requiring mechanical ventilation; shock state; and patients with other types of organ failure that need to be monitored in the ICU. This study was approved by the Institutional Ethics Committee of Renmin Hospital of Wuhan University.

### 2.2 Data collection

Demographic characteristics, clinical records, laboratory data, ECG characteristics, treatments, and outcome data were obtained with data collection forms from electronic medical records. Two experienced clinicians entered and reviewed the data. Recorded information included demographic data, medical history, symptoms and signs, chronic diseases, laboratory findings, ECG data, and treatment measures. The date of disease onset was defined as the day when the symptom was noticed. The criteria for a confirmed diagnosis of SARS‐CoV‐2 were that at least one gene site was amplified and considered positive for the nucleocapsid protein (NP) gene and open reading frame (ORF) gene.^9^ Myocardial injury was defined as blood levels of cardiac biomarkers (hs‐TnI) above the 99th‐percentile upper reference limit, regardless of new abnormalities in electrocardiography and echocardiography.^4^ QT prolongation was defined as an absolute QTc interval > 500 ms (or a JTc interval > 410 ms to adjust for patients with QRS duration >120 ms).^10^

### 2.3 Outcomes

The outcomes were death or discharge. Discharge standards were defined according to the guidance of the New Coronavirus Pneumonia Prevention and Control Program in China (sixth edition).^8^

### 2.4 Statistical analysis

Categorical variables are expressed as numbers (%) and were compared by Pearson's Chi‐square test or Fisher's exact test. Univariate analysis was used to evaluate demographics and clinical factors associated with COVID‐19 mortality. We used Kaplan‐Meier survival analysis to estimate the patient survival fraction and the stratified log‐rank test to compare the difference in survival distributions between different groups. Time to events (death) was defined as the time from illness onset to events. Two groups were created, 'death' and 'recovery', to study the relationship between high‐risk factors and mortality from COVID‐19 using hazard ratios (HRs) generated by a Cox proportional hazards regression model. A forward selection procedure was then used to construct an initial model. Based on clinical experience, a final model was selected. Proportional hazards assumptions were systematically verified for the proposed models. Hypothesis testing was conducted using a two‐sided test, and an alpha value of 0.05 indicated statistical significance. A forest plot was created based on multivariate Cox regression results. All analyses were performed using the SPSS (version 20.0) and GraphPad Prism 8.0.

## 3 RESULTS

### 3.1 Baseline characteristics

A total of 113 patients with critical COVID‐19 were included in this study; 68 were male, and 45 were female. The mean age was 67.3 ± 14.1 years, ranging from 29 to 95 years. 50 patients (44.3%) died, and 63 (55.7%) recovered before March 15, 2020. The median length of stay was 17 days (IQR, 5‐28 days). The median length of stay of the death group was 4 days (IQR, 3‐7 days), and in the recovery group, the median length of stay was 26 days (IQR, 22‐39 days). Baseline characteristics of the 113 confirmed cases are shown in (Supplemental Table S1). There was a significant difference among age groups (>70 years and < 70 years) and clinical outcomes (death and recovery) (*P* = .008). Hypertension (*P* = .042) and temperature greater than 39°C (*P* = .039) were more common in patients who died. There were no significant differences in sex, chronic diseases, (such as, diabetes, cerebrovascular disease, COPD, chronic kidney disease and chronic liver disease), or initial symptoms, (such as, fever, cough, fatigue, anorexia, myalgia, dyspnea, pharyngalgia, diarrhea, vomiting, and dizziness) between the death group and recovery group.

### 3.2 Laboratory findings

As shown in Table 1, the following factors were associated with a high risk of death from COVID‐19: white blood cell count greater than 9.5 × 10^9^/L (*P* = .001), initial neutrophil count greater than 6.5 × 10^9^/L (*P* < .001), initial lymphocyte count less than 0.6 × 10^9^/L (*P* = .011), C‐reactive protein greater than 100 mg/L (*P* < .001), D‐dimer greater than 20 mg/L (*P* = .003), hypersensitive troponin I greater than 0.04 pg/mL (*P* = .004), blood urea nitrogen greater than 8 mmol/L (*P* = .011), lactate dehydrogenase greater than 300 U/L (*P* < .001), and lactic acid greater than 3 mmol/L (*P* = .014). However, there were no differences in hemoglobin less than 120 g/L, platelet count less than 100 × 10^9^/L, procalcitonin greater than 0.5 ng/mL, creatine kinase‐MB greater than 5 ng/mL, alanine aminotransferase greater than 50 U/L, aspartate aminotransferase greater than 40 U/L, albumin less than 30 g/L, creatinine greater than 100 μmol/L, creatine kinase greater than 200 U/L, and B‐type natriuretic peptide (BNP) greater than 900 pg/mL between the death group and recovery group.

**TABLE 1:** Characteristics of laboratory results in patients with COVID‐19

Laboratory results	No.(%)			*P*‐value
	All cases (n = 113)	Death cases (n = 50)	Recovery cases (n = 63)	
White blood cell count>9.5 × 10^9^/L	22(19.47)	17(34.00)	5(7.94)	.001
Initial neutrophil count>6.5 × 10^9^/L	38(33.63)	28(56.00)	10(15.87)	<.001
Initial lymphocyte count<0.6 × 10^9^/L	44(38.94)	26(52.00)	18(28.57)	.011
Hemoglobin<120 g/L	49(43.36)	20(40.00)	29(46.03)	.520
Platelet count<100 × 10^9^/L	18(15.93)	11(22.00)	7(11.11)	.116
C‐reactive protein>100 mg/L	44(38.94)	31(62.00)	13(20.63)	<.001
Procalcitonin>0.5 ng/mL	21(18.58)	12(24.00)	9(14.29)	.187
D‐dimer>20 mg/L	15(13.27)	12(24.00)	3(4.76)	.003
Creatine kinase‐MB > 5 ng/mL	15(13.27)	8(16.00)	7(11.11)	.447
Hypersensitive troponin I > 0.04 pg/mL	38(33.63)	24(48.00)	14(22.22)	.004
Alanine aminotransferase>50 U/L	39(34.51)	17(34.00)	22(34.92)	.919
Aspartate minotransferase>40 U/L	57(50.44)	29(58.00)	28(44.44)	.152
Albumin<30 g/L	21(18.58)	12(24.00)	9(14.29)	.187
Blood urea nitrogen>8 mmol/L	44(38.94)	26(52.00)	18(28.57)	.011
Creatinine>100 μmol/L	20(17.70)	11(22.00)	9(14.29)	.286
Creatine kinase>200 U/L	24(21.24)	14(28.00)	10(15.87)	.117
Lactate dehydrogenase>300 U/L	73(64.60)	42(84.00)	31(49.21)	<.001
BNP > 900 pg/mL	39(34.51)	21(42.00)	18(28.57)	.136
Lactic acid>3 mmol/L	34(30.09)	21(42.00)	13(20.63)	.014

Abbreviation: BNP, brain natriuretic peptide.

### 3.3 Characteristics of ECG outcomes

ECG data were available for 70 patients, of whom 35.7% died and 64.3% survived. Table 2 shows the ECG characteristics. Ventricular arrhythmias were recorded in 8 patients. In the death group, there were 5 cases of premature ventricular contraction and 1 case of ventricular tachycardia. In the recovery group, 2 patients had premature ventricular contraction. The proportion of patients with ventricular arrhythmia was higher in the death group than in the recovery group (24.0% vs 4.4%; *P* = .021) and was higher among myocardial damage patients than nonmyocardial damage patients (26.1% vs 4.3%; *P* = .013). There was a significant difference in abnormal ECG results between the death group and recovery group (*P* = .041). However, there was no significant difference in ST‐T abnormalities between patients with and without myocardial damage. Furthermore, other arrhythmic events, such as, sinus tachycardia, atrioventricular block, and atrial arrhythmia, showed no difference between the death group and recovery group. There was no significant difference in the QT interval between patients with and without hydroxychloroquine treatment.

**TABLE 2:** Characteristics of ECG outcome with the study population

ECG characteristics	No.(%) Myocardial damage cases (n = 23)	Non‐myocardial damage cases (n = 47)	*P*‐value	No.(%) Death cases (n = 25)	Recovery cases (n = 45)	*P*‐value
Abnormal ECG	18(78.26)	27(57.45)	.088	20(80.00)	25(55.56)	.041
Abnormal ST‐T	11(47.83)	16(34.04)	.266	12(48.00)	15(33.33)	.227
Anterior ST‐T changes	5(21.74)	2(4.26)		4(16.00)	3(6.67)	
Inferior ST‐T changes	3(13.04)	1(2.13)		2(8.00)	2(4.44)	
All lead ST‐T changes	3(13.04)	13(27.66)		6(24.00)	10(22.22)	
Prolonged QT	4(17.39)	6(12.77)	.719^a^	5(20.00)	5(11.11)	.477^a^
Sinus tachycardia	5(21.74)	4(8.51)	.143^a^	6(24.00)	3(6.67)	.060^a^
Sinus bradycardia	0(0)	3(6.38)	.546^a^	0(0)	3(6.67)	.548^a^
Atrioventricular block	3(13.04)	2(4.26)	.322^a^	3(12.00)	2(4.44)	.341^a^
RBBB	1(4.35)	2(4.26)		2(8.00)	1(2.22)	
LBBB	2(8.70)	0(0)		2(8.00)	0(0)	
First degree A‐V block	2(8.70)	0(0)		1(4.00)	1(2.22)	
Pathological Q wave	3(13.04)	2(4.26)	.322^a^	3(12.00)	2(4.44)	.341^a^
Atrial arrhythmia	5(21.74)	2(4.26)	.035^a^	3(12.00)	4(8.89)	.694^a^
Atrial premature beat	2(8.70)	1(2.13)		1(4.00)	2(4.44)	
Atrial tachycardia	1(4.35)	0(0)		1(4.00)	0(0)	
Atrial fibrillation	2(8.70)	1(2.13)		1(4.00)	2(4.44)	
Ventricular arrhythmia	6(26.09)	2(4.26)	.013^a^	6(24.00)	2(4.44)	.021^a^
PVC	5(21.74)	2(4.26)		5(20.00)	2(4.44)	
Ventricular tachycardia	1(4.35)	0(0)		1(4.00)	0(0)	

*Note*: one case combined with first degree A‐V block, complete RBBB, left anterior fascicular block and prolonged QT.

Abbreviations: ECG, electrocardiogram; LBBB, left bundle branch block; PVC, premature ventricular contraction; RBBB, right bundle branch block.

^a^ Fisher's exact test.

### 3.4 Treatment characteristics

Table 3 shows that the proportion of patients with Arbidol (82.5% vs 58.0%; *P* = .004) and hydroxychloroquine treatment (23.8% vs 4.0%; *P* = .003) was higher in the recovery group than in the death group. Other antiviral drugs, such as lopinavir/ritonavir, ribavirin, interferon α‐2b injection, ganciclovir, and oseltamivir, showed no difference between the death group and recovery group. In addition, glucocorticoid therapy, immunoglobulin, albumin therapy, oxygen therapy, noninvasive ventilation (NIV), and invasive mechanical ventilation (IMV) were not significantly different between critical patients in the death group and recovery group.

**TABLE 3:** Characteristics of treatment with the study population

Treatment	No.(%)			*P*‐value
	All cases (n = 113)	Death cases (n = 50)	Recovery cases (n = 63)	
Antiviral drug				
lopinavir/ritonavir	4(3.54)	2(4.00)	2(3.17)	1.000^a^
ribavirin	56(49.56)	23(46.00)	33(52.38)	.500
arbidol	81(71.68)	29(58.00)	52(82.54)	.004
hydroxychloroquine	17(15.04)	2(4.00)	15(23.81)	.003
interferon α‐2b injection	21(18.58)	9(18.00)	12(19.05)	.887
ganciclovir	20(17.70)	11(22.00)	9(14.29)	.286
oseltamivir	34(30.09)	18(36.00)	16(25.40)	.222
Glucocorticoid therapy	70(61.95)	30(60.00)	40(63.49)	.704
Immunoglobulin	73(64.60)	29(58.00)	44(69.84)	.191
Albumin therapy	27(23.89)	10(20.00)	17(26.89)	.387
Oxygen therapy	53(46.90)	22(44.00)	31(49.21)	.582
NIV	56(49.56)	26(52.00)	30(47.62)	.644
IMV	4(3.54)	2(4.00)	2(3.17)	1.000^a^

Abbreviations: IMV, invasive mechanical ventilation; NIV, noninvasive ventilation.

^a^ Fisher's exact test.

### 3.5 Risk factors associated with death

Kaplan‐Meier survival analysis was used to analyze patient survival. Supplemental Figure S1 shows the survival curves of patients of different ages (<70 years and >70 years). Elderly patients were more common in the death group than in the recovered group (*P* = .009). The survival curve of those who had an initial neutrophil count >6.5 × 10^9^/L was lower than that of patients with an initial neutrophil count <6.5 × 10^9^/L (*P* < .001) (Supplemental Figure S2). The survival curve of patients with C‐reactive protein >100 mg/L was lower than that of patients with C‐reactive protein <100 mg/L (*P* < .001) (Supplemental Figure S3). The survival curve of patients with lactate dehydrogenase >300 U/L was lower than that of patients with lactate dehydrogenase <300 U/L (*P* < .001) (Supplemental Figure S4). Immunoglobulin therapy was more common in the recovered group than in the death group (*P* = .227) (Supplemental Figure S5).

All the factors in Tables S1, 1 and 3 were included in multivariate analysis to explore independent predictors of mortality from COVID‐19. As there were only 70 ECG data points, the factors in Table 2 were used in multivariate analysis alone to explore only the ECG risk factors for mortality from COVID‐19. As show in Table 4, the independent mortality predictors of COVID‐19 were age >70 years (HR 1.84, 95% CI 1.03‐3.28), initial neutrophil count greater than 6.5 × 10^9^/L (HR 3.43, 95% CI 1.84‐6.40), C‐reactive protein greater than 100 mg/L (HR 1.93, 95% CI 1.04‐3.59), and lactate dehydrogenase greater than 300 U/L (HR 2.90, 95% CI 1.26‐6.67), which were all distributed to the right of the invalid line, as shown in Supplementary Figure S6. Immunoglobulin treatment (HR 0.39, 95% CI 0.21‐0.73) reduced the risk of death and was distributed to the left of the invalid line in the forest plot. In Table 5, sinus tachycardia (HR 2.94, 95% CI 1.16‐7.46) and ventricular arrhythmia (HR 2.79, 95% CI 1.11‐7.04) were independent ECG risk factors for mortality from COVID‐19.

**TABLE 4:** Cox proportional hazards regression model of risk factors for COVID‐19

Characteristics	coefficient	SE (coefficient)	wald	*P*‐value	HR(95%CI)
Age >70 years	0.608	0.295	4.246	.039	1.84(1.03‐3.28)
Initial neutrophil count>6.5 × 10^9^/L	1.232	0.319	14.930	<.001	3.43(1.84–6.40)
C‐reactive protein>100 mg/L	0.656	0.318	4.272	.039	1.93(1.04–3.59)
Lactate dehydrogenase>300 U/L	1.063	0.425	6.249	.012	2.90(1.26–6.67)
Immunoglobulin treatment	−0.935	0.320	8.568	.003	0.39(0.21–0.73)

Abbreviations: CI, confidence interval; HR, hazard ratio.

**TABLE 5:** Cox proportional hazards regression model of ECG risk factors for COVID‐19

Characteristics	Coefficient	SE (coefficient)	wald	*P*‐value	HR(95%CI)
Sinus tachycardia	1.077	0.476	5.126	.024	2.94(1.16‐7.46)
Ventricular arrhythmia	1.027	0.471	4.745	.029	2.79(1.11‐7.04)

## 4 DISCUSSION

This present retrospective study identified several risk factors for mortality from COVID‐19. In particular, old age (>70 years), neutrophilia, C‐reactive protein greater than 100 mg/L and lactate dehydrogenase greater than 300 U/L were associated with a higher likelihood of critical in‐hospital death. Our study also showed that the incidence of ventricular arrhythmia was higher in deceased patients than survivors. Sinus tachycardia and ventricular arrhythmia were independent ECG risk factors for mortality from COVID‐19. However, there was no difference in ST‐T and QT interval abnormalities between deceased patients and survivors.

In slightly over 3 months, SARS‐CoV‐2 spread worldwide and caused far greater morbidity and mortality than either SARS or MERS.^11^ Previous studies have shown that older age, D‐dimer greater than 1 μg/mL and greater cardiac troponin are potential risk factors for inpatients with COVID‐19.^12^, ^13^ The number of cases has rapidly increased throughout the world, and there are more severe cases. However, the risk factors for death are not fully understood in critical cases. In the present study, we analyzed possible risk factors for death from COVID‐19. All patient characteristics and laboratory findings were included to examine the relationship between risk factors and death from critical COVID‐19 at an early stage. The risk factors related to death included older age, neutrophilia, C‐reactive protein greater than 100 mg/L, and lactate dehydrogenase greater than 300 U/L. Chen suggested that SARS‐CoV‐2 is more likely to infect older adult males with chronic comorbidities as a result of the weaker immune functions of these patients.^2^ We also found that the proportion of elderly patients and hypertension patients was higher in patients who died. Therefore, as an independent risk factor, age‐related chronic diseases still play an important role in the outcome of critical cases. In addition, the results of the present study showed that patients with COVID‐19 who died had significantly higher neutrophil counts than survivors. Considering that older age is associated with decreased immune function,^14^ older age may be related to death due to less robust immune responses.

Cytokine storm and the viral evasion of cellular immune responses are thought to play important roles in disease severity.^15^ The present findings showed that CRP greater than 100 mg/L was significantly associated with fatality. A significant increase in CRP levels, as documented for bacterial infections, can also occur with viral infections.^16^ CRP is a classic acute phase protein. It can be concluded that a higher CRP value may result from a more severe form of COVID‐19. Lactate dehydrogenase greater than 300 U/L is another independent high‐risk factor for mortality. Increased lactate dehydrogenase was related to heart failure or MOF, which may lead to the fatality from COVID‐19.^17^

SARS‐CoV‐2 infection is associated with inflammatory mediators that may play important roles in cardiac and arrhythmic complications.^18^, ^19^, ^20^ A previous study reported that 16.7% of patients with COVID‐19 had arrhythmia, and 7.2% had acute heart injury.^6^ However, other studies have reported that the incidence of arrhythmia in patients with COVID‐19 was only 0.3%, which was relatively low.^21^, ^22^ In our study, to the best of our knowledge, we first reported ECG characteristics in critical patients. We found that the incidence of arrhythmia was approximately 45.7% in critical patients and that the incidence of ventricular arrhythmia was high in patients with myocardial damage and in patients who died. Sinus tachycardia and ventricular arrhythmia were independent ECG risk factors for mortality in critical inpatients with COVID‐19. Furthermore, there was no difference in the QT interval between patients who died and survivors, and there was no significant difference in ST‐T abnormalities between patients with and without myocardial damage. Taken together, these results showed that critical inpatients with COVID‐19 are prone to ventricular arrhythmia or abnormal ECG results, which is caused by myocardial damage.

Hydroxychloroquine is known to have anti‐inflammatory and antiviral effects and is used for rheumatoid arthritis and SARS.^23^, ^24^ The side effects of hydroxychloroquine may include gastrointestinal symptoms and QT prolongation syndrome, especially in patients with renal or hepatic dysfunction.^25^ However, our results showed that hydroxychloroquine treatment was not associated with a higher likelihood of survival in critical in‐hospital patients. Furthermore, hydroxychloroquine treatment during hospitalization was not associated with QT prolongation.

There were several limitations to this study. First, most of the patients did not have a 24‐hour Holter monitor. Short bursts of arrhythmias may have been missed. Second, few patients were given antiarrhythmic drugs, such as, amiodarone and propafenone. Whether antiarrhythmic drugs affect the occurrence of arrhythmia needs further study. Third, due to the retrospective study design and the limited number of patients, data from larger populations and multiple centers are needed to further confirm the risk of mortality during hospitalization. Finally, this was a retrospective and observational study, and most of the patients were seriously ill at the time of admission. Very few patients had echocardiographic data, and patient height and weight data were also missing, so we could not obtain results of echocardiography and BMI.

## 5 CONCLUSIONS

Old age (>70 years), neutrophilia, C‐reactive protein greater than 100 mg/L, and lactate dehydrogenase greater than 300 U/L are high‐risk factors related to the fatality of critical patients with COVID‐19. Immunoglobulin treatment can reduce the risk of death. The proportion of patients with ventricular arrhythmia was higher in deceased patients than in survivors. Sinus tachycardia and ventricular arrhythmia were independent ECG risk factors for mortality in critical inpatients with COVID‐19.

## CONFLICT OF INTEREST

The authors declare no potential conflict of interest.

## AUTHOR CONTRIBUTIONS

Lingzhi Li, Shudi Zhang, and Bing He conducted the systematic literature search, analyzed data and wrote the manuscript. Xiaobei Chen and Shihong Wang revised the manuscript for intellectual content. Qingyan Zhao is the guarantor of this work and, as such, had full access to all the data in the study and takes responsibility for the integrity of the data and the accuracy of the data analysis

## Supporting information


**Appendix S1. Supporting Information**
