# Supplementary material for: Risk factors and electrocardiogram characteristics for mortality in critical inpatients with COVID‐19
Source: Clin Cardiol. 2020 Oct 22;43(12):1624–30. doi: 10.1002/clc.23492 (PMC7724237; doi:10.1002/clc.23492)
Supplement: Supplementary file 1 — Appendix S1. Supporting Information [file CLC-43-1624-s001.docx]

**Supplementary Table S1**

Baseline characteristics of the study population

| Baseline characteristics | No.(%) |  |  | P-value |
| --- | --- | --- | --- | --- |
|  | All cases  (n=113) | Death cases  (n=50) | Recovery cases  (n=63) |  |
| Sex  male  female  Age(yrs)  >70  Chronic diseases  hypertension  cardiovascular disease  diabetes  cerebrovascular disease  COPD  chronic kidney disease  chronic liver disease  malignancy  First symptom  fever  temperature>39℃  cough  fatigue  anorexia  myalgia  dyspnea  pharyngalgia  diarrhea  vomiting  dizziness | 68(60.18)  45(39.82)  52(46.02)  49(43.36)  23(20.35)  21(18.58)  8(7.08)  12(10.62)  6(5.31)  4(3.54)  5(4.42)  104(92.04)  60(53.10)  69(61.06)  50(44.25)  48(42.48)  11(9.73)  69(61.06)  11(9.73)  18(15.93)  5(4.42)  8(7.08) | 33(66.00)  17(34.00)  30(60.00)  27(34.00)  14(28.00)  9(18.00)  5(10.00)  7(14.00)  3(6.00)  2(4.00)  2(4.00)  47(94.00)  32(64.00)  30(60.00)  25(50.00)  24(48.00)  5(10.00)  31(62.00)  7(14.00)  7(14.00)  2(4.00)  3(6.00) | 35(55.56)  28(44.44)  22(34.92)  22(34.92)  9(14.29)  12(19.05)  3(4.76)  5(7.94)  3(4.76)  2(3.17)  3(4.76)  57(90.48)  28(44.44)  39(61.90)  25(39.68)  24(38.10)  6(9.52)  38(60.32)  4(6.35)  11(17.46)  3(4.76)  5(7.94) | 0.260  0.008  0.042  0.072  0.887  0.463^a^  0.299  1.000^a^  1.000^a^  1.000^a^  0.729^a^  0.039  0.837  0.273  0.290  1.000^a^  0.855  0.211^a^  0.618  1.000^a^  1.000^a^ |

^a^ Fisher’s exact test.

**Supplementary Figure S1** Survival plot for patients over 70 years old (green line) compared to patients who younger than 70 years old (blue line), duration since illness onset.


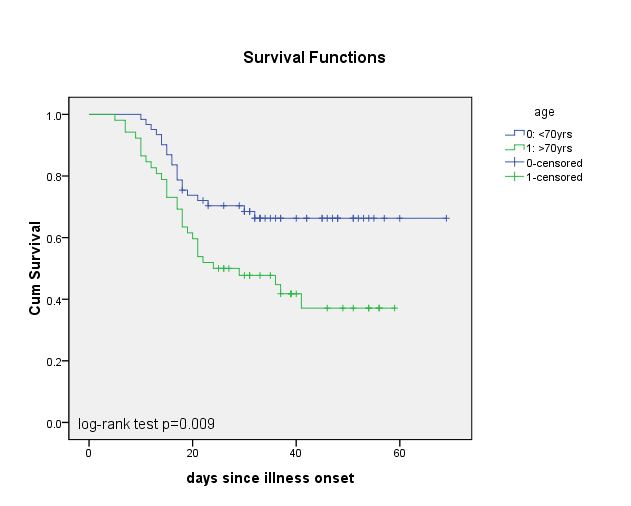


**Supplementary Figure S2** Survival plot for patients with initial neutrophil count > 6.5×10^9^/L (green line) compared to patients with initial neutrophil count < 6.5×10^9^/L (blue line), duration since illness onset.


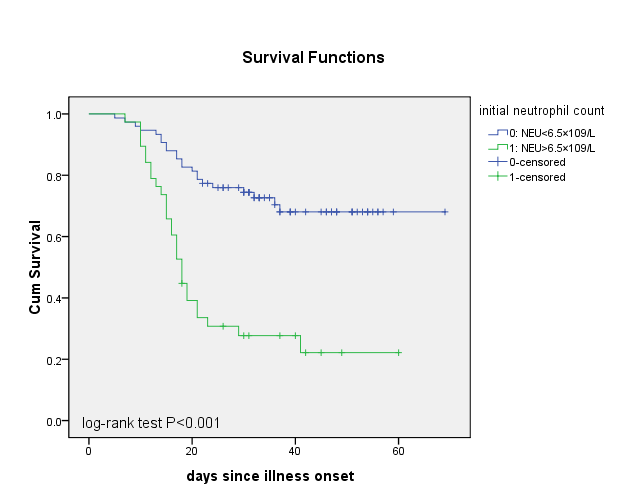


**Supplementary Figure S3** Survival plot for patients with C-reactive protein > 100mg/L (green line) compared to patients with C-reactive protein < 100mg/L (blue line), duration since illness onset.


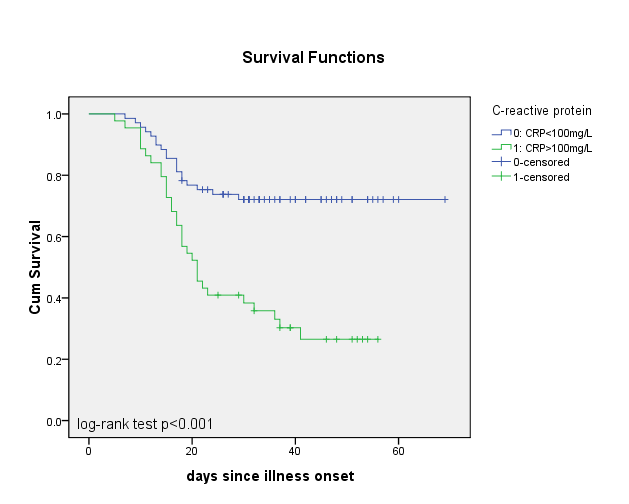


**Supplementary Figure S4** Survival plot for patients with lactate dehydrogenase > 300U/L (green line) compared to patients with lactate dehydrogenase < 300U/L (blue line), duration since illness onset.


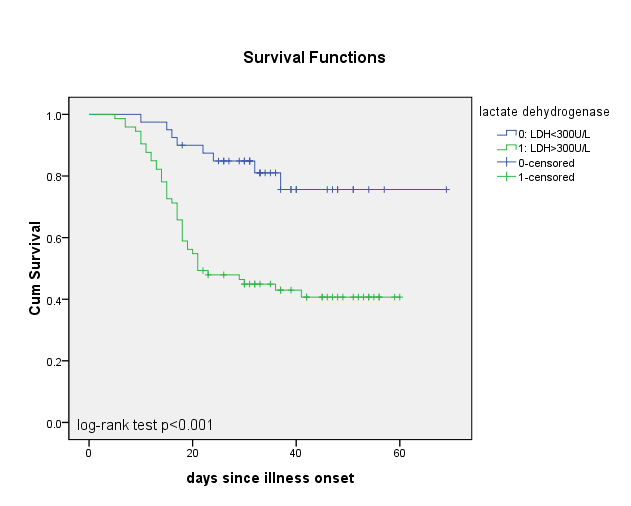


**Supplementary Figure S5** Survival plot for patients with immunoglobulin treatment (green line) compared to patients without immunoglobulin treatment (blue line), duration since illness onset.


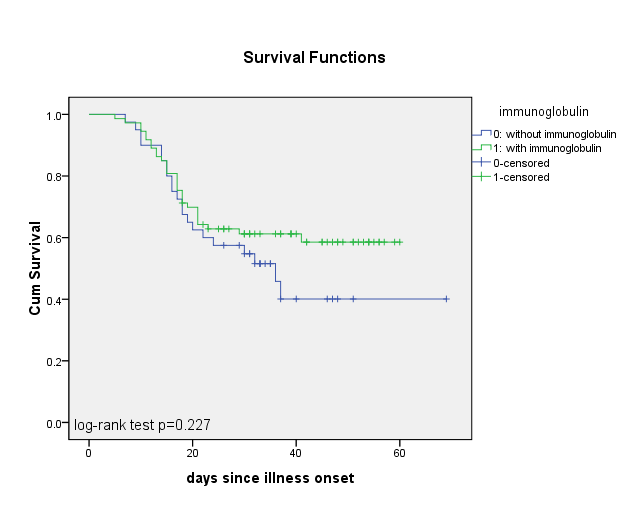


**Supplementary Figure S6** the forest plot of factors associated with mortality of critical COVID-19 by Cox regression analysis.

**
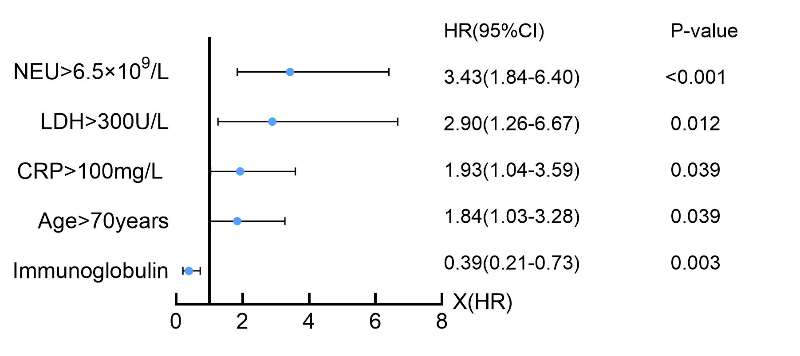
**
